# Visual Analysis of College Sports Performance Based on Multimodal Knowledge Graph Optimization Neural Network

**DOI:** 10.1155/2022/5398932

**Published:** 2022-07-01

**Authors:** Nan Zheng, Meng Sun, Ye Yang

**Affiliations:** ^1^School of Physical Education and Training, Shanghai University of Sport, Shanghai 200438, China; ^2^College of Sports and the Arts, Hebei University of Sport, Shijiazhuang, Hebei 050049, China; ^3^School of Continuing Education, Shanghai University of Sport, Shanghai 200438, China

## Abstract

In this paper, through data analysis of multimodal knowledge graph optimized neural network and visual analysis of college students' sports performance, we use huge graph, a graph database supporting distributed storage, to store domain knowledge in the form of the knowledge graph, use Spring Boot to build a server-side framework, use Vue framework combined with vis.js to visualize relational network graphs, and design and implement a knowledge-oriented. This paper proposes a visual analytics system based on the theory of visual analytics. Based on the idea of visual analytics, this paper presents a visual analytics framework combining predictive models. This framework combines the automated analysis capability of predictive models with interactive visualization as a new idea to explore the visual analysis of student behavior and performance changes. Using relevant predictive algorithms in machine learning, corresponding models are built to refine the importance of features for visual analysis and correlate behavioral data with achievement data. In this process, multiple prediction algorithms are used to build prediction models. The model effects are analyzed and compared to select the optimal model for use in the visual analytics framework. The graphical analytic view is integrated. EduRedar, an optical analytical system for sports data based on the performance prediction model, is designed and implemented to support multidimensional and multiangle data analysis and visualize the changes in college students' sports and performance based on accurate campus exercise data.

## 1. Introduction

With the widespread use of computer technology in various industries, Internet technology has hugely impacted traditional physical education and research. The physical education sector particularly values data analysis of college students' sports performance because the study of sports performance data is one of the critical topics for researchers in the sports sector. However, due to the limitation of professional knowledge, it is not easy to discover the explicit phenomena and tacit knowledge contained in sports performance data with a large data volume [1]. They need a tool that can visually demonstrate the characteristics of various aspects of the data to help them process it, preferably by presenting it in images. It is necessary to develop a physical test score management system using computer-related techniques to improve efficiency significantly [2]. It is also helpful to use data mining and machine learning techniques to visualize images with data analysis capabilities and detect anomalies, efficiently solving complex research and daily work problems.

Knowledge graph (KG) provides an efficient data organization and management model. It has become a hot research problem in recent years, and its related technologies and applications have made significant progress [3]. Currently, two essential types of knowledge graph construction are general-purpose knowledge graph (GKG) construction and domain-specific knowledge graph (DKG) construction. The available domain knowledge graph construction data mainly come from significant databases and search sites and are built top-down. A usable open knowledge network is finally obtained through entity analysis and extraction of comprehensive knowledge, with typical examples, such as Wikidata, DBpedia, and Knowledge Vault. Due to the characteristics of the extensive knowledge scale and high complexity of GKG, it is often challenging to meet the needs of users in practical applications [4]. The construction of a vertical domain knowledge graph is generally oriented in a specific direction, firstly, through industry data analysis, determining the extraction target, selecting the extraction method, and using the extraction results to build a large-scale domain semantic network. Typical DKGs include Microsoft's academic knowledge graph and Leetaru's geopolitical event graph. In the constructed DKGs, knowledge inference in the graphs and logical correctness verification can be easily realized because the professional relevance between knowledge is more substantial and more closely connected. However, most of the existing studies on the construction of vertical domain knowledge graphs focus on unimodal charts (e.g., text graphs and image graphs), ignoring the critical significance of multimodal data such as audio, video, and image in the era of big data and the essential characteristics of multimodal data. There are fewer research works on the construction of graphs for many multimodal data. In this paper, we focus on domain multimodal knowledge management, carry out research on multimodal knowledge graph construction, construct domain multimodal data sets, propose a fusion model of multimodal data (text, image, and video), complete the construction of multimodal knowledge graph for a specific domain, and develop a corresponding validation system. The multimodal fusion theoretical framework proposed in this paper has theoretical significance for solving the problems such as the semantic gap between multimodal data and can provide reference models for related research work. The constructed experimental system for intelligent applications has practical significance for intelligent retrieval and knowledge Q&A intelligence.

Knowledge graph can extract and classify the massive information on the Internet, visually present the structural relationship of knowledge points and the distribution of course knowledge points, simplify information access, reveal the knowledge structure, and help knowledge discovery. Visualization technology can describe knowledge resources and their carriers, mine, analyze, construct, draw, and display knowledge [5]. The structural relationship between them is the source of knowledge mapping construction and deep thinking. Knowledge mapping can form a transparent knowledge system and efficiently facilitate learners to acquire high-quality and practical knowledge. Compared with concept maps, knowledge maps, and cognitive maps, knowledge maps can characterize the overall system of a specific discipline and contain a broader range of knowledge, a large amount of knowledge and its relationships. From the perspective of knowledge scope, concept maps, knowledge maps, and cognitive maps, all describe only a topic in a discipline and contain a narrow range of knowledge, with more straightforward knowledge content and relationships. From the perspective of relationship type, the relationship type of concept map is usually only the relationship between whole and part, a single type [6]. The relationship types of cognitive maps are usually cause-effect relationships. In contrast, the relationship type of knowledge maps is a semantic relationship, which can relate relationships through semantics. Thus, knowledge mapping can express a broader range of knowledge contents and semantic association relationships than concept maps, knowledge maps, and cognitive maps.

## 2. Related Works

As the use of computer technology becomes more and more abundant and better understood, computer technology is increasingly used to assist in the management of the work of various industries. Computer-aided management can significantly improve efficiency and reduce manual labor because of the multiple tasks involved in the teaching and learning process [7]. As a normal part of the educational management in colleges and universities, the sports performance management system can significantly reduce tedious and complicated manual work by using computer technology to process the results. The scholar Zheng et al. used the combination of VB programming language and SQL library to design and build the sports performance management system, which contains some functional modules in the management of the system [8]. The plan includes available modules in this management, which satisfies the daily management of grades in higher education institutions. Feng et al. proposed using structured analysis and design theory to build a sports performance management system for universities to meet the needs of physical education and performance management [9].

In contrast to the above two scholars' research, Munir et al.'s research is broader and more involved, pointing out that they use ASP.NET technology and client-side programming languages Javascript and VBScript to create a college sports performance management system developed through the B/S model [10]. This is because the data structure plays a vital role in the statistics and analysis of the results. The use of formal methods to build the database model of the system paves the way for later query functions and statistical analysis [11]. In visualizing sports performance data, Chanson et al. used the SOM and PCA algorithms in machine learning to visualize sports performance data and studied the geographical characteristics of sports performance [12]. Huang et al. cheated out the knowledge graph of sports performance and gave the research hotspots and frontier trends in sports performance through visualization techniques [13]. Although a few studies on the visualization of sports performance data are mentioned above, the studies on visualization of images in more sports performance data management systems are more conventional and do not explore the problem of missing items and anomalies in sports performance data in depth. Dhillon et al. conducted data visualization of students' use of learning management system (LMS) based on extensive data analysis framework, which helped nonbig data student management practitioners in the field improve the efficiency of analyzing the data [6]. Based on their analysis, they identified the positive effects of the LMS on student learning and made recommendations to students and instructors based on their findings. This system allows us to analyze the students' thinking when solving programming problems and provide suggestions for the instructor's teaching methods. In addition to visual analysis of online courses, there is also an analysis of students' psychological state [14].

Knowledge mapping is an emerging metrological research method developed based on citation analysis theory and information technology [15]. It visualizes the development process and structural relationship of scientific knowledge through data mining, information processing, measurement, and graphical drawing, reveals scientific expertise and activity law, and shows the structural relationship and evolution law of expertise [16]. It consists of theories of graphology, information technology, statistics, metrology, citation analysis, and co-occurrence analysis. It follows the principles of Kuhn's theory of scientific development patterns, Price's theory of scientific frontiers, structural hole theory of social network analysis, information foraging theory of scientific communication, theory of discrete and reorganization of knowledge units, etc., to extract data from large samples of academic information such as journal papers, foundation project information, research topics, and keywords and perform clues. It also provides visualization of the structure, relationship, and evolution process of the knowledge in the academic science field, showing the core structure, development history, frontier areas, and overall knowledge structure of the discipline and providing a practical and valuable reference for the discipline research [17].

## 3. Multimodal Knowledge Graph Optimization Neural Network Model Construction

Depending on the data sources, the knowledge graph can be constructed in two ways: top-down and bottom-up. The former refers to defining the ontology and data schema for the knowledge graph first and then extracting specific instances from high-quality data to add to the knowledge base; for example, data can be obtained from structured data sources such as encyclopedic websites. They needed a tool to help them process the data by visually displaying all aspects of the data's characteristics, preferably in graphic form. The latter extracts entities and relationships from open data, selects information with higher confidence to add to the knowledge base, and then constructs the top-level ontology schema [18]. Since the bottom-up approach makes better use of the massive resources on the Internet, it has gradually become the mainstream knowledge graph construction method. The main steps in knowledge graph construction are entity recognition and relation extraction. Web applications eliminate the tedious steps of installation and maintenance of client applications for users. They can be accessed by a browser only while also providing the same powerful and complete services as client applications, significantly reducing the cost of application usage. Named entity recognition refers to recognizing entities with specific meanings from texts, such as names of people, places, organizations, and proper nouns. Named entity recognition is a fundamental task in natural language processing, and its accuracy will affect the effectiveness of downstream tasks.

**Figure 1:**
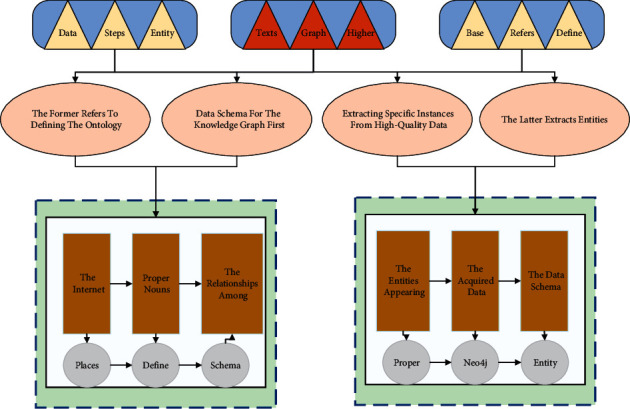
Construction framework of multimodal course knowledge map.

On the contrary, entity-relationship extraction is based on the semantic information of the text and inferring the relationships among the entities appearing in the text. In this paper, we first define the data schema of the multimodal course knowledge graph and then complete the work of named entity identification and relationship classification in the acquired data, followed by multimodal entity connection. Finally, the extracted entities and relationships are stored in the graph database Neo4j to complete the knowledge graph visualization. The construction framework of the multimodal course knowledge graph is shown in Figure 1.

The methods in the construction of multimodal knowledge graphs include data collection and processing, schema construction, multimodal knowledge extraction, multimodal knowledge fusion, graph visualization, and graph application. The methods used in multimodal knowledge fusion are calculating similarity, label alignment, and data linking. Data visualization is the process of presenting raw data in a visual form which is then recognized by the human cognitive system. The visualization and analysis method can also be understood as similar to the process of coding and decoding. The database for building multimodal knowledge graphs in this paper is text, image, and video data from the related websites of Baidu.com. Suppose these multisource heterogeneous data are used directly. In that case, the subsequent construction and application will be significantly affected because of the different organization of the data and some unusable fragmented data, so these multisource heterogeneous data should be preprocessed first to ensure the essential correctness of the subsequent process.

Neural networks are an essential component structure of many machine learning models, such as deep learning and graph neural networks, and play a vital role in multimodal feature learning. In the process of feature extraction of multimodal data using neural networks, it is necessary to select appropriate neural network features for feature extraction according to the characteristics of different modal data, for example, using a feature extractor based on convolutional neural networks for image feature extraction and using a feature extractor based on recurrent neural networks for time-series data, such as text and sensors. The perceptron is the primary constituent structure of the neural network with a set of N-dimensional feature vectors *X*=[*x*_1_, *x*_2_,…, *x*_*n*_], weights in the network *W*=[*w*_1_, *w*_2_,…, *w*_*n*_], bias b, and an activation function *f*(*∗*), and finally, the output of the perceptron *h*:

(1) $$\begin{array}{c} f\left(z\right)=\sum_{i=1}\frac{wx_{i}+N}{b}. \end{array}$$

**Figure 2:**
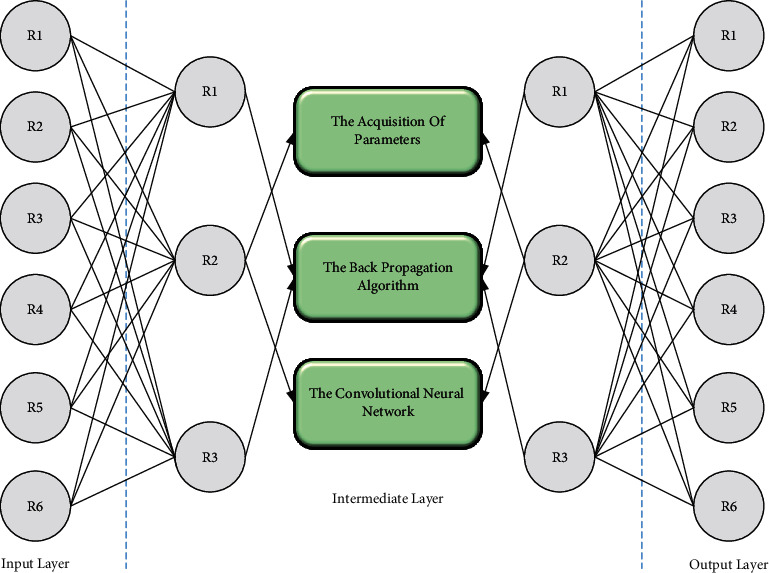
Multilayer perceptron.

The perceptron operates by weighting and summing the input feature vectors and obtaining the output feature *h* through the activation function *f*=*w∗x* − *b*. The output feature *h* can be further input to the next layer of the perceptron, and the multilayer perceptron (MLP) structure is obtained through multiple similar operations, as shown in Figure 2. The input layer of the perceptron network is a linear transformation function to get linear features. Then, a nonlinear activation function is introduced to obtain the nonlinear relationship between the elements. For the acquisition of parameters in the perceptron and multilayer perceptron, the training of the model is mainly achieved by the backpropagation algorithm ( BP), which requires not only the sample input but also the sample labels to calculate the output loss function and the gradient of the output layer [19]. Then, the chain derivative rule calculates the angle of each layer in the multilayer perceptron and uses the slope to update its rise, which is then used to calculate the gradient of each layer in the multilayer perceptron. The angle is used to update the parameters of each layer to complete the training of the model.

For image features, spatial features are essential in images, and multilayer perceptrons ignore spatial information. In addition, high-dimensional features like images can significantly increase the computation of multilayer perception. Therefore, a convolutional neural network (CNN) is proposed to extract multidimensional edge features in photos. The computational effort of the model is significantly reduced by sharing weights, local connections, etc. The computation of convolution is suitable for parallel computation to improve the computational efficiency of the model. Compared to the most basic network structure of neural networks, the fully connected layer (MLP), where the feature matrix is multiplied by the weight matrix, and the graph neural network has an additional adjacency matrix. The computational form is simple; three matrices and a nonlinear transformation are born. The convolution operation in the convolutional neural network can be understood as the filtering operation of the image, i.e., the core of the convolutional network algorithm is to use the convolutional kernel to perform the sliding operation in the picture, and the adjacent pixels sliding through the convolutional kernel are weighted and summed to obtain the output of the convolution. The edge features of different dimensions in the image can be extracted by setting additional sliding steps. The idea still retains the original spatial, the painting has its original spatial features after the convolution operation, and the convolutional features can be input to the next convolutional layer. Although the depth of the convolutional neural network can improve the ability of the model to extract features, the increase in the number of layers of the network, gradient disappearance, gradient explosion, and other problems make the network cannot be trained, so ResNet was proposed to solve the problem of gradient disappearance in deep convolutional neural networks, which mainly uses the residual network structure to ensure that the features of the previous layer can be smoothly passed to the next layer. The convolutional neural network with the residual module can stack the convolutional layers very profoundly and then train them with a large amount of labeled data to achieve high accuracy in image classification tasks. In addition, the features extracted from the middle layer of the pretrained convolutional neural network can be used for functions related to multimodal feature learning, such as target detection and image annotation:

(2) $$\begin{array}{c} x_{l}=\sum_{l=1}\frac{\sqrt{h\left(x-l\right)}}{x_{1}+w_{1}}. \end{array}$$

The input features of neural networks are independent of each other. Many serialized data in multimodal data have backward and forward dependence on the input, so recurrent neural networks (RNN) are proposed for feature extraction of such data. The recurrent neural network differs from the perceptron. The information of the recurrent neural network includes not only the current input but also the hidden layer features of the previous state. The recurrent function is calculated by equation (3), where *f*(*∗*) denotes the nonlinear activation function, *g*(*∗*) is the linear function or SoftMax function, *h* represents the state of the hidden layer, *o* is the output of the recurrent neural network, and *w*_*xh*_, *w*_*xh*_, and *w*_*xy*_ are the model training parameters:

(3) $$\begin{array}{c} h_{t}=\int \frac{\sqrt{w_{xh}x+w_{xh}}}{\sqrt{w_{yh}+h_{t}}}. \end{array}$$

Graph convolutional neural networks are essential for graph structure data feature extraction. In multimodal feature learning graphs, neural networks apply to modeling topological relationship graphs within each modality and topological relationships between multiple modalities, so they have an essential role in multimodal feature learning. The graph neural network based on spectral analysis is one of the most common types of neural graph networks, and its central idea is the propagation of features from neighboring nodes:

(4) $$\begin{array}{c} h_{i}=\sum g\left(h_{1}+h_{j}\right). \end{array}$$

## 4. Visual Analysis Model Design for College Students' Sports Performance

**Figure 3:**
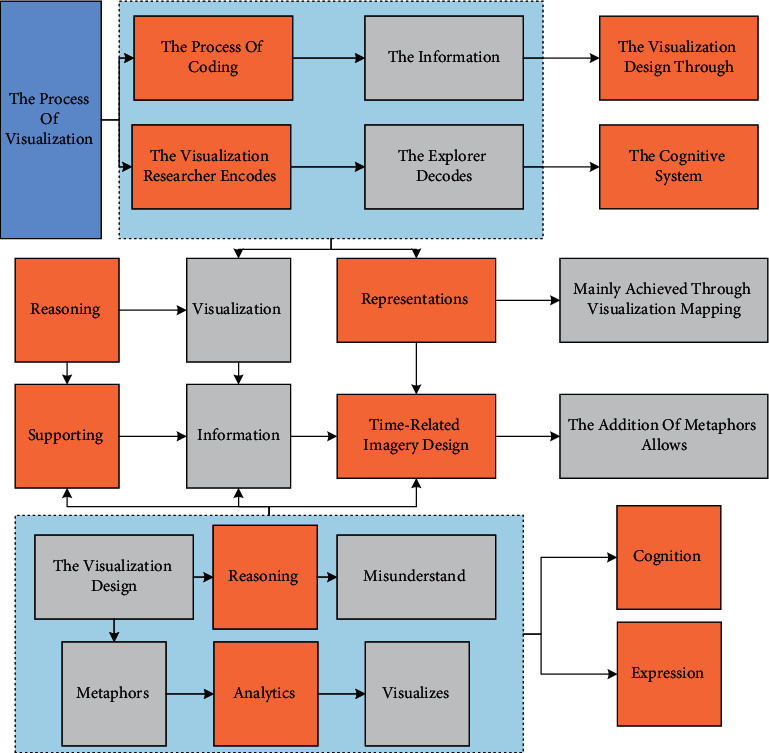
Visual analysis system structure diagram.

Data visualization is the process of presenting raw data in a visual form that is then recognized by the human cognitive system. The visualization and analysis method can also be understood as coding and decoding: the researcher encodes the information, and the explorer decodes the data from the visualization design through the cognitive system. Transforming raw data into encoding for visualization is mainly achieved through visualization mapping. Visualization mapping is data mapping to graphical images that the human visual system can perceive. Commonly used visual representations are spatial location and graph attributes such as shape and size. Data visualization researchers need to meet two basic requirements when creating visual mappings: (1) the visual mapping must not change the data to ensure that the data are accurate and (2) the representation or metaphor must not be obscure and needs to be quickly understood by the user. Easy to understand requires data visualization designers to be able to design with the full expression of data features in mind and the common sense of human cognition. Visualization designers need to refer to the needs of the target users before designing the visualization, which can be used to guide the visualization design. When the data structure is complex, particular attention needs to be paid to the simplicity of the visualization design. Intricate visualization design will make it difficult for users to understand the data and even make them misunderstand the data [20]. For complex data, the method can be presented hierarchically by adding interactive means and according to a certain logic, thus making the view design easy to understand and improving users' understanding efficiency. When designing data visualization, using metaphors that conform to human cognition helps express data information efficiently. Just the fitting metaphor can also add artistry to the visual analytics design. For example, when coding time-related knowledge, time-related imagery design can be used. A metaphor like a mind map is utilized when visualizing data for analyzing process and management hypothesis evidence. The addition of metaphors allows for an intuitive and comprehensive grasp of the reasoning process in the analysis process while also supporting rapid tracking to a particular node. The trade-off between practicality and artistry also needs to be determined by the goals of the data visualization. Based on these four proposed design requirements, we have specifically divided the system design into three modules: the analysis of the model's module, the analysis of the group performance module, and the analysis of the individual performance module. The analysis module of the model shows the results of the prediction model and visualizes the features used in the prediction model; the analysis module of group performance supports the exploration of group performance from different time dimensions; the analysis module of individual performance allows focusing on individual performance and analyzing individual students' physical activity. The structure diagram of the visual analysis system is shown in Figure 3.

**Figure 4:**
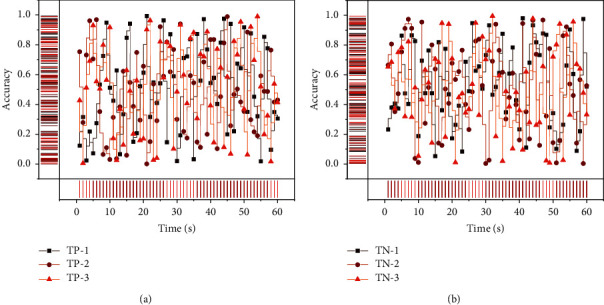
Comparison chart of model accuracy.

The visual analytics design requirements require visualization of the results of the prediction model, as well as the display of the essential features filtered through the prediction model. Therefore, in the visualization of the prediction model, we designed the student-type sunburst diagram to display the prediction results. For feature presentation, feature parallels coordinate view and feature heat map are intended to support the multidimensional exploration of features. According to the results of the behavioral prediction model, students can be classified into four categories: “TP is correctly predicted as a student in the yellow zone,” “TN is correctly predicted as a student in the nonyellow zone,” “FN is predicted as nonyellow line students are yellow line zone students,” and “FP is predicted as yellow line students are nonyellow line zone students.” In the design basis of visual analysis, the model results are the basis for screening, and these four categories of students will be used as the starting point for visual research to explore the behavioral characteristics of each category of students. In the model analysis module, what needs to be presented are the four categories of students obtained based on the predictive model, the characteristics selected in the predictive model, and the characteristics of high importance obtained from the model. The classification results of the predictive model will be presented in the student-type sunburst diagram. The prediction results are divided into four categories, and the students in each category can be further classified by grade, college, etc. Based on such a hierarchical structure, a design that can show the hierarchical structure needs to be used. To display the effect of the classification algorithm, the percentage of samples in each category of the prediction results needs to be shown. Based on the two considerations of the hierarchical structure and the percentage situation, we use the design of the rising sun chart [21]. The sunburst chart is based on a pie chart with an additional hierarchical structure consisting of multiple layers of rings. From the center of the circle outward, the percentage of each type in each level is coded by area, and different types are distinguished by color. The story of the center of the circle shows the percentage of students in the four classes. By analyzing the portion of the four types, we can have a preliminary grasp of the model's accuracy. If the area of two items of the model, TP and TN, is more extensive and the size of FP and FN is smaller, it means that the model has a better classification effect. The comparison chart of the model accuracy is shown in Figure 4.

After designing and implementing the visual analytics view, we need to integrate the idea to implement a complete visual analytics system. This section will introduce the technical dependencies of the visual analytics system EduRedar. With the development of web technology and the advent of the web 2.0 era, web applications are increasingly widely used. For users, web applications eliminate the tedious steps of installing and maintaining client applications and can be accessed by a browser only while also providing the same powerful and complete services as client applications, significantly reducing the cost of using the application. The development of web servers and the new trend of front-end and back-end decoupling empower independent front-end development reduce the burden of back-end servers, which significantly improves the efficiency of developers and lowers development costs. In this context, B/S architecture-based web applications are blossoming, and developers are exploring more possibilities for web applications.

**Figure 5:**
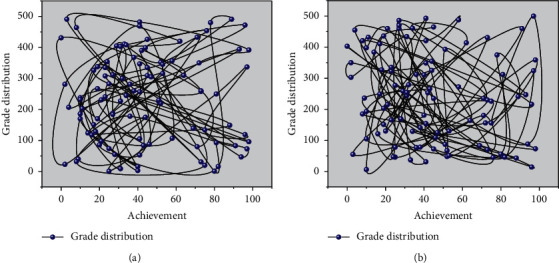
Distribution of students' actual sports performance.

The results of our analysis of physical education scores are consistent with the experience of our counselors: high and low student performance in mathematics can reflect, to some extent, students' academic performance. Therefore, we added the midterm and final grades of Calculus I and linear algebra in the first academic year as features in the prediction model [22]. Considering the experience of judging students in actual exercise and the distribution of students' physical education grades, as shown in Figure 5, we chose 70 as the division point. We defined the “yellow line students” quantitatively as those whose weighted average scores of all required courses were lower than 70 at the end of the sixth semester. If stored in a single table, it will be because of the size of a single table and then produce the problem of the high cost of adding, deleting, changing, and checking operations. If stored in separate tables, many joint processes between multiple tables will also be time-consuming and have low performance. Thus, the prediction model can be summarized as a dichotomous problem of predicting whether a student's weighted average score will be less than 70 in the sixth semester for a student's feature vector. Four algorithms for solving the dichotomous problem are tried in this study, namely, logistic regression (LR), random forest (RF), gradient boosting decision tree (GBDT), and GBDT + LR algorithm. This study will find the best prediction algorithm from these four algorithms to obtain the importance of features for visual analysis.

## 5. Analysis of Results

### 5.1. Multimodal Knowledge Graph Optimization Neural Network Model Analysis

The multimodal feature interaction process is a long process of extracting data. As the model needs, this section will use different methods to process the model features and finally perform the interaction input to the model. First, a modified xDeep FM model that can accept multimodal features as information is shown, specifying how to integrate all the modified models to form a deep fusion model [23]. Based on xDeep FM, some modules are added to extract grades and sports performance features. Firstly, sparse user interaction data need to be converted to light features; then, dense user interaction data need to be converted to thick parts; secondly, title data need to be converted to sequence features; finally, an embedding layer is used to reduce the dimensionality of these features and obtain the embedded feature vector. The result of the embedding layer is a wide cascade vector:

(5) $$\begin{array}{c} e=\oint \left(\frac{e_{1}}{2}+\frac{e_{2}}{e_{m}}\right), \end{array}$$

where *m* denotes the number of features and *e*_*i*_ ∈ *R*^*d*^ represents the *i*th element's embedding vector; these embedded feature vectors are fed into three modules: linear layer, compressed interaction network (CIN) layer, and deep neural network (DNN) layer. The input of the CIN layer comes from the embedding layer, and assuming that there are *m* features and the embedding vector dimension of each element is *D*, the input can be represented as a matrix *x*° ∈ *R*^*M*×*d*^, such that *x*^*k*^ ∈ *R*^*k*×*d*^ denotes the output of the *k*th layer, where *H*_*k*_ means the *k*th layer vector. The number of vectors and the vector latitude is *D*, which is kept consistent with the input layer. Specifically, each vector in the *k*th layer is computed as

(6) $$\begin{array}{c} K_{h}=\sum_{i=1}w_{ij}\left(x_{i}^{k}+x_{j}\right). \end{array}$$

For sports performance data, firstly, principal component analysis (PCA) is used to extract the main components of the original features and reduce the feature size; secondly, the whitening operation is used to reduce the correlation between elements, and then, an embedding layer is used to obtain the embedded feature vectors of sports performance features; finally, the embedded sports performance feature vectors and sports performance data feature vectors are fed into two different pure DNN layers for further feature extraction. It not only performs higher-order feature learning of implicit and explicit multimodal features and converts feature interactions into trainable vector values but also has the breadth and remarkable learning properties. The final output result equation is

(7) $$\begin{array}{c} y=\sum \delta \frac{w_{\text{linear}}}{w_{\text{dnn}}+x_{\text{dnn}}}-w_{\text{lcin}}p. \end{array}$$

**Figure 6:**
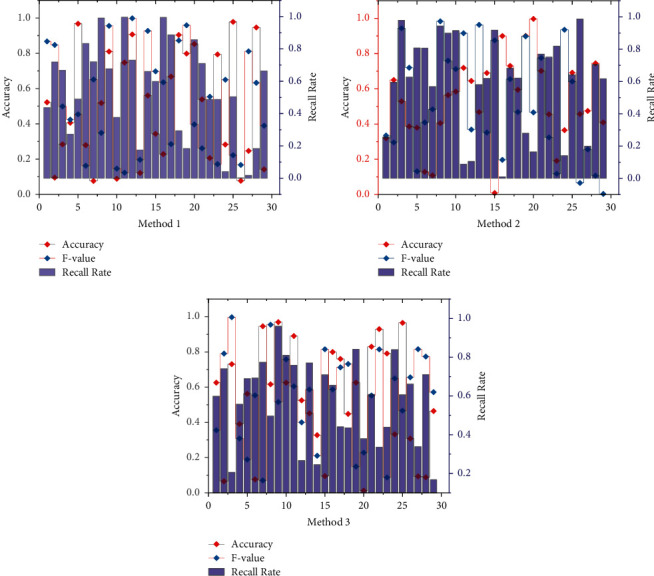
Comparison of recognition effects of three named entity recognition methods.

The data of the knowledge map are usually represented in the form of <entity, relation, entity> triples, which can be stored in various ways, either in text file format, such as RDF files, or in a database. Because of the continuous development of the discipline, the content of the curriculum knowledge map will be constantly adjusted. New knowledge units and relationships will be created, and knowledge units and associations will be extinguished due to the aging of information resources. Res Net was proposed to solve the problem of gradient disappearance in deep convolutional neural networks, which mainly used a network structure of residuals to ensure that the features of the previous layer can be passed to the next layer smoothly. Therefore, static text files are not conducive to updating knowledge mapping data, and it is more appropriate to use databases for storage. Databases are divided into traditional relational databases and nonrelational databases. When storing knowledge graphs, conventional relational databases store one triad. The graph database is a kind of nonrelational database, which is specially optimized for the storage and query of graph structure, and the data type is in the form of node and relationship connection; this data storage method makes their data access performance and question efficiency greatly improved compared with the traditional relational database [24]. In addition, it can also draw on the relevant algorithms of graph theory to further deep mining and reasoning of knowledge. Therefore, graph databases are widely used in the development process of personalized recommendation systems and knowledge graph construction systems. A comparison of the recognition effects of the three named entity recognition methods is shown in Figure 6.

### 5.2. Visualization and Analysis of College Students' Sports Performance

This section compares the recommended performance metrics of different models on different recommended data, and the recommended performance metrics used in this section are Recall@20 and NDCG@20, where the optimal number of convolutional layers of the relationship graph is used for the recommended results of KGMRCF, and the specific recommended results are shown in Table 1. Among the models compared in the table, FM and NFM are both factorization-based algorithms whose basic principle is to use feature vectors to compute inner products to obtain multiorder feature relations; CKE and CFKG are regularity-based algorithms, i.e., the loss function of Trans R is used as the standard term in the model loss function; Ripple Net is an algorithm that combines graph path and regularity; GC-MC is a GCN based algorithm. The experimental results show that the recommendation model KGMRCF proposed in this section outperforms the traditional algorithm and similar knowledge graph recommendation algorithms in various recommendation performance indexes, but due to the variability of the recommendation dataset, in which the dataset ranking on the interaction record is Last-FM > Yelp2018 > Amazon-Book in order, which is consistent with the collaborative filtering algorithm in the data volume. This is in line with the fundamental principle that the collaborative filtering algorithm is more accurate in recommending results when the amount of data is significant. The order of datasets in terms of the number of relationships in the knowledge graph is Yelp2018 > Amazon-Book > Last-FM. Regarding the number of samples and connections, the Last-FM dataset has the most significant number of training samples and the smallest number of relationships, so it is easier for KGMRCF to learn from more data. Therefore, the KGMRCF model outperforms KGAT by five percentage points in the Last-FM dataset regarding recommendation performance. In addition, the experimental results on the Amazon-Book recommendation dataset show that the version of KGMRCF is closer to that of the KGAT algorithm, which is because the relationship attribute of Amazon-Book contains more extensive domain knowledge. The data volume is smaller, so it cannot thoroughly learn the related knowledge in the domain. Therefore, the recommendation performance of KGMRCF on the sparse knowledge graph dataset is closer to that of the Yelp2018 dataset which is a restaurant recommendation dataset, and the knowledge graph relational attribute information of this dataset is mainly the facilities and functions of some restaurant merchants, etc. These relational attribute features have greater relevance to users' preferences [25]. There are more implicit correlations, so compared with KGAT, KGMRCF can better obtain users' implicit relational features, so KGMRCF has 2–3 percentage points higher recommendation performance than KGAT.

We try to use LR, RF, GBDT, and GBDT + LR algorithms to select the best prediction algorithm and refine the data in the prediction model. In this paper, we used K-fold cross-validation. In terms of samples and number of relations, the Last-FM dataset has the most significant number of training samples and the smallest number of ties, so it is easier for KGMRCF to learn the characteristic relations between different relations from more data. Hence, the recommendation performance of the KGMRCF model in the Last-FM dataset is better than KGAT by as much as 5 percentage points. We utilized the standard classification metrics of binary classification problems, namely, precision, recall, and *F*1-score, to compare the effectiveness of the models. The GBDT + LR algorithm is slightly better than GBDT and RF, but in realistic terms, the goal of prediction is to find the “yellow line students” more accurately, so the recall of the algorithm is more demanding. Therefore, after weighing the performance of the GBDT, RF, and GBDT + LR algorithms, we chose the GBDT + LR results as our basis for classifying students into four categories: “TP is correctly predicted as yellow zone students,” “TN is correctly predicted as nonyellow zone students,” and “TN iscorrectly predicted as nonyellow zone students.” “ “FN ispredicted as nonyellow line students are yellow line zone students” and “FP is predicted to be yellow line students are non-yellow line students” for visual analysis of college athletic performance.

**Figure 7:**
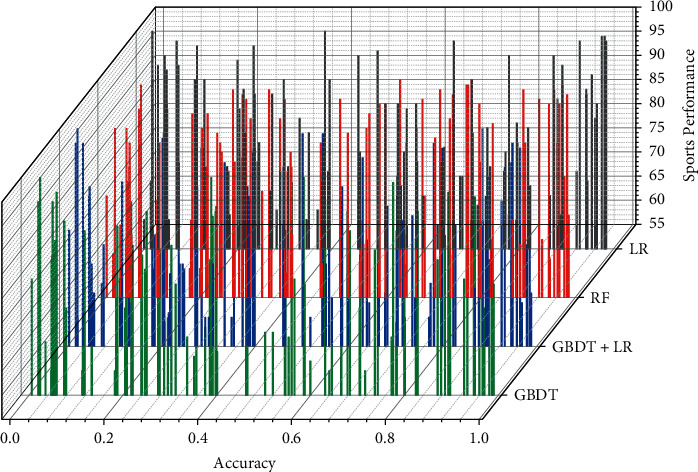
Visual comparison chart of sports performance.

The experiments show that all modified models accurately predict the test set. The prediction accuracy of the models in this section is higher than the other baseline models in finish and like, with values of 0.7315 and 0.9356, respectively, and in score, with a value of 0.7936. In the finish comparison, the deep FM model performs the worst, with a value of 0.7276, and XDeep FM serves the best. In the like comparison, the FNN model performs the worst in like with a value of 0.9204 and the NFFM model performs the best with a value of 0.9287, which is 0.7% higher than the best baseline model. In the score comparison, the PNN model performs the worst with a value of 0.77748 and the NFM model performs the worst with a value of 0.7936. The NFM model performed the worst with a value of 0.78925, and the model in this section was 0.5% higher than the best baseline model. The visualization of the sports performance comparison is shown in Figure 7.

## 6. Conclusion

In this paper, based on the actual requirements of knowledge graph visualization and analysis, the system is analyzed and designed in detail, the overall technical architecture and functional modules are determined, the graph database huge graph is used to store the knowledge graph. Huge graph underlying storage supports plug-in multiple storage configurations, the system adopts HBase as the storage side, and a distributed storage cluster environment is built to ensure the system's extensive data storage requirements and reliability. An excellent visual analysis view is designed for the prediction model results and behavior data. In this paper, we analyze the college students' sports performance, derive the target of visual analysis based on this analysis, and formulate the design basis of the graphical view. According to the design basis, the visual analysis view is divided into model analysis, group performance analysis, and individual performance. The visualization mapping, layout algorithm, and interaction method are designed to realize the polar coordinate scatter diagram, calendar spreads chart, and behavioral river diagram to explore the visual analysis view of college students' sports performance in different time dimensions. By integrating the visual analysis views of three modules, a model analysis, group performance analysis, and individual performance, we design and implement EduRedar, an optical analysis system of college students' sports performance data based on an exercise prediction model, which supports the multilevel and multiangle exploration of the data. The system supports the multilevel and multiperspective exploration of the data. It can understand the whole picture of the data from a macroperspective and focus on individuals for a detailed analysis of college students' sports performance.

## Figures and Tables

**Table 1:** Comparison of recommended performance indicators for each model.

Algorithm	NDCG	RECALL	NDCG	RECALL	NDCG	RECALL
Improve	0.3812	0.7833	0.1968	0.0874	0.6225	0.1218
KGMRCF	0.1934	0.9952	0.9255	0.4668	0.0024	0.3882
KGAT	0.2722	0.8166	0.2758	0.9713	0.9929	0.8275
GC-MC	0.2430	0.9505	0.1684	0.8261	0.1227	0.4573
RIPPLENET	0.9995	0.4045	0.7950	0.1971	0.0155	0.4727
CFKG	0.1312	0.3075	0.8879	0.0644	0.3158	0.8443
CKE	0.1958	0.6186	0.3652	0.8062	0.8976	0.0545
NFM	0.0416	0.7960	0.8265	0.4523	0.3221	0.7747
FM	0.4216	0.4838	0.8838	0.2773	0.7735	0.5256

## Data Availability

The data used to support the findings of this study are available from the corresponding author upon request.
